# The genome sequence of the Yellow-legged Gull, *Larus michahellis *Naumann, 1840

**DOI:** 10.12688/wellcomeopenres.23849.1

**Published:** 2025-03-14

**Authors:** Elisa Ramos, Manuel Schweizer, Meng Yue Wu, Christophe Sahli, Constantin Latt, Maurice Lunak, Pierre-André Crochet, Walter Salzburger, Joana Meier, David Alexander Marques, Wellcome Sanger Institute Tree of Life Management, Samples and Laboratory team, Wellcome Sanger Institute Scientific Operations: Sequencing Operations, Wellcome Sanger Institute Tree of Life Core Informatics team, Tree of Life Core Informatics collective, Darwin Tree of Life Consortium

**Affiliations:** 1 Natural History Museum Basel, Basel, Switzerland; 2 Zoological Institute, Department of Environmental Science, University of Basel, Basel, Switzerland; 3 Natural History Museum Bern, Bern, Switzerland; 4 Institute of Ecology and Evolution, University of Bern, Bern, Switzerland; 5 Association de la Grande Caricaie, Cheseaux-Noréaz, Vaud, Switzerland; 6 CEFE/CNRS, Montpellier, France; 7 Tree of Life, Wellcome Sanger Institute, Hinxton, England, UK

**Keywords:** Larus michahellis, Yellow-legged Gull, genome sequence, chromosomal, Charadriiformes

## Abstract

We present a genome assembly from a female specimen of *Larus michahellis* (Yellow-legged Gull; Chordata; Aves; Charadriiformes; Laridae). The genome sequence has a total length of 1,405.56 megabases. Most of the assembly (90.55%) is scaffolded into 35 chromosomal pseudomolecules, including the W and Z sex chromosomes. The mitochondrial genome has also been assembled and is 16.79 kilobases in length.

## Species taxonomy

Eukaryota; Opisthokonta; Metazoa; Eumetazoa; Bilateria; Deuterostomia; Chordata; Craniata; Vertebrata; Gnathostomata; Teleostomi; Euteleostomi; Sarcopterygii; Dipnotetrapodomorpha; Tetrapoda; Amniota; Sauropsida; Sauria; Archelosauria; Archosauria; Dinosauria; Saurischia; Theropoda; Coelurosauria; Aves; Neognathae; Neoaves; Charadriiformes; Laridae; *Larus*; *Larus michahellis* J.F.Naumann, 1840 (NCBI:txid119627)

## Background

The Yellow-legged Gull ( *Larus michahellis*) is an adaptable species of seabird commonly breeding around the Mediterranean Sea and Iberia, with its range extending to the Southern half of the Black Sea in the East, to the UK and inland Central Europe up to Poland in the North, to Morocco in the South and to the Canary Islands, Madeira and Azores in the West ( del Hoyo *et al.*, 2020). It has undergone considerable range expansion North- and Eastwards during the 20th and early 21th century thanks to improved protection of breeding sites and access to food through fisheries and landfills ( Hagemeijer & Blair, 1997; Keller *et al.*, 2020; Tsvelykh, 2022), and is currently listed as 'Least Concern' by the IUCN with a continuing positive population trend ( BirdLife International, 2019). While Western populations are sedentary, Central European and Eastern populations are partial migrants showing an unusual migration pattern by moving Northwest to the Atlantic coasts of Central and West Europe between July and October before returning to their breeding grounds in late winter ( Olsen & Larsson, 2010).

**Figure 1.**
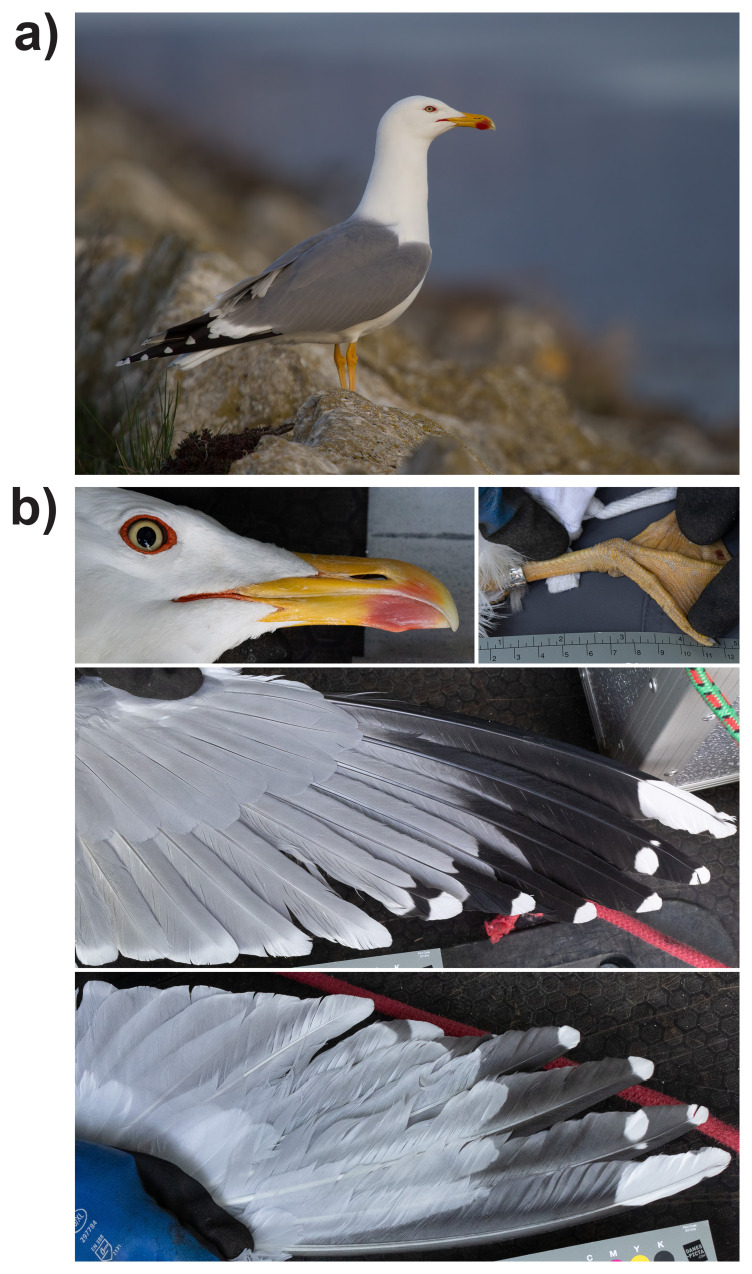
**a**) Yellow-legged Gull ( *Larus michahellis*), adult female, 15 April 2018, Cudrefin VD, Switzerland. **b**) Photographs of the female Yellow-legged Gull ( *Larus michahellis*, MA02451, NMB-AVES-23-001) from which samples were taken for sequencing. Note the red gonys bleeding into the upper mandible, red gape and orange-red eye ring, clean yellow iris, deep yellow legs, complete black band across the fifth primary and large extent of black in the outer primaries.

Long considered either a subspecies or colour polymorphism within European Herring Gull *L. argentatus*, the Yellow-legged Gull received full species status in the early 2000s following genetic studies ( Crochet *et al.*, 2002). Adult Yellow-legged Gulls ( Figure 1a) are characterised by deep yellow legs, grey upperparts slightly darker blue-grey than European Herring Gull, a dark orange to red eye ring, gape and large red gonys spot on the beak, a pale iris and a primary pattern featuring much black, including most of the outermost primary and a broad band on the fifth primary counted outwards ( Olsen & Larsson, 2010). Today, two or sometimes three subspecies of *L. michahellis* are generally recognised: *L. m. michahellis* (Western and Southern Europe, Northwest Africa, and the Mediterranean north to Poland and east to Turkey), *L. m. atlantis* Dwight, 1922 (Azores, Madeira and Canary Islands), and *L. m. lusitanus* Joiris, 1978 (Northwestern Iberia between Penice and the Basque Country). The latter is often included within *L. m. michahellis* ( Olsen & Larsson, 2010; Romero *et al.*, 2019). Following its range expansion, *L. michahellis* is known to have hybridised when coming in contact with other, parapatric large white-headed gull species, particularly *L. cachinnans*, *L. argentatus*, and *L. fuscus* ( Olsen & Larsson, 2010). However, hybridisation is considered to be rare nowadays where those species breed in sympatry and thus considered of limited taxonomic significance ( Neubauer *et al.*, 2010), even though it raises questions on what phenotypic or genomic barriers contribute to reproductive isolation and which genes crossed species barriers during hybridisation ( Pons *et al.*, 2004).

Breeding colonies are found primarily on sea cliffs, rocky islands, inside coastal wetlands and more recently on roofs of buildings ( Olsen & Larsson, 2010). Following other gull species, the Yellow-legged Gull has successfully colonised urban areas and is increasingly becoming commensal in parts of its range, with an increasing and expanding population of birds now preferring to nest on rooftops in towns and seaports ( Benussi & Fraissinet, 2020). The species' diet has been extensively studied in Mediterranean areas, where *L. michahellis* primarily feeds on fish and marine invertebrates, but also exploits anthropogenic food sources ( Lopes *et al.*, 2021). The ingestion of anthropogenic debris, particularly microplastics, poses significant concerns for their health and the environment, as microplastics have been found in the faeces and regurgitated pellets of *L. michahellis* in Spain, Portugal, and France, highlighting their potential as vectors of microplastic pollution among others to protected sites ( Ceia *et al.*, 2014; Galimany *et al.*, 2023; Lopes *et al.*, 2021; Nono Almeida *et al.*, 2023; Senes *et al.*, 2023).

## Genome sequence report

### Sequencing data

The genome of a specimen of *Larus michahellis* ( Figure 1b) was sequenced using Pacific Biosciences single-molecule HiFi long reads, generating 87.57 Gb from 8.71 million reads. GenomeScope analysis of the PacBio HiFi data estimated the haploid genome size at 1,239.01 Mb, with a heterozygosity of 0.45% and repeat content of 13.02%. These values provide an initial assessment of genome complexity and the challenges anticipated during assembly. Based on this estimated genome size, the sequencing data provided approximately 68.0x coverage of the genome. Chromosome conformation Hi-C data produced 290.44 Gb from 1,923.41 million reads. Table 1 summarises the specimen and sequencing information, including the BioProject, study name, BioSample numbers, and sequencing data for each technology.

**Table 1.** Specimen and sequencing data for *Larus michahellis*.

Project information			
**Study title**	Larus michahellis (yellow-legged gull)		
**Umbrella BioProject**	PRJEB76381		
**Species**	*Larus michahellis*		
**BioSpecimen**	SAMEA114211328		
**NCBI taxonomy ID**	119627		
Specimen information			
**Technology**	**ToLID**	**BioSample ** **accession**	**Organism ** **part**
**PacBio long read sequencing**	bLarMic1	SAMEA114211357	oviduct
**Hi-C sequencing**	bLarMic1	SAMEA114211380	blood
**RNA sequencing**	bLarMic1	SAMEA114211380	blood
Sequencing information			
**Platform**	**Run accession**	**Read count**	**Base count ** **(Gb)**
**Hi-C Illumina NovaSeq X**	ERR13248997	6.26e+08	94.56
**Hi-C Illumina NovaSeq X**	ERR13248998	7.75e+08	116.96
**Hi-C Illumina NovaSeq X**	ERR13248999	5.23e+08	78.91
**PacBio Revio**	ERR13245309	8.71e+06	87.57
**RNA Illumina NovaSeq X**	ERR13248992	6.23e+07	9.4
**RNA Illumina NovaSeq X**	ERR13248991	5.14e+07	7.76
**RNA Illumina NovaSeq X**	ERR13248993	5.05e+07	7.63
**RNA Illumina NovaSeq X**	ERR13248995	5.69e+07	8.6
**RNA Illumina NovaSeq X**	ERR13248996	6.21e+07	9.38
**RNA Illumina NovaSeq X**	ERR13248994	4.05e+07	6.12

### Assembly statistics

The primary haplotype was assembled, and contigs corresponding to an alternate haplotype were also deposited in INSDC databases. The assembly was improved by manual curation, which corrected 69 misjoins or missing joins and removed 1 haplotypic duplications. These interventions decreased the scaffold count by 5.79%, and increased the scaffold N50 by 3.52%. The final assembly has a total length of 1,405.56 Mb in 617 scaffolds, with 500 gaps, and a scaffold N50 of 87.71 Mb ( Table 2).

**Table 2.** Genome assembly data for *Larus michahellis*.

Genome assembly		
Assembly name	bLarMic1.1	
Assembly accession	GCA_964199755.1	
*Alternate haplotype * *accession*	*GCA_964199725.1*	
Assembly level for primary assembly	chromosome	
Span (Mb)	1,405.56	
Number of contigs	1,117	
Number of scaffolds	617	
Longest scaffold (Mb)	224.38	
Assembly metrics	Measure	*Benchmark*
Contig N50 length	4.22 Mb	*≥ 1 Mb*
Scaffold N50 length	87.71 Mb	*= chromosome N50*
Consensus quality (QV)	Primary: 63.8; alternate: 64.1; combined: 63.9	*≥ 40*
*k*-mer completeness	Primary: 94.01%; alternate: 84.30%; combined: 99.81%	*≥ 95%*
BUSCO *	C:97.4%[S:96.9%,D:0.6%], F:0.4%,M:2.2%,n:8,338	*S > 90%* *D < 5%*
Percentage of assembly mapped to chromosomes	90.15%	*≥ 90%*
Sex chromosomes	W and Z	*localised * *homologous pairs*
Organelles	Mitochondrial genome: 16.79 kb	*complete single * *alleles*

* BUSCO scores based on the aves_odb10 BUSCO set using version 5.4.3. C = complete [S = single copy, D = duplicated], F = fragmented, M = missing, n = number of orthologues in comparison.

**Figure 2.**
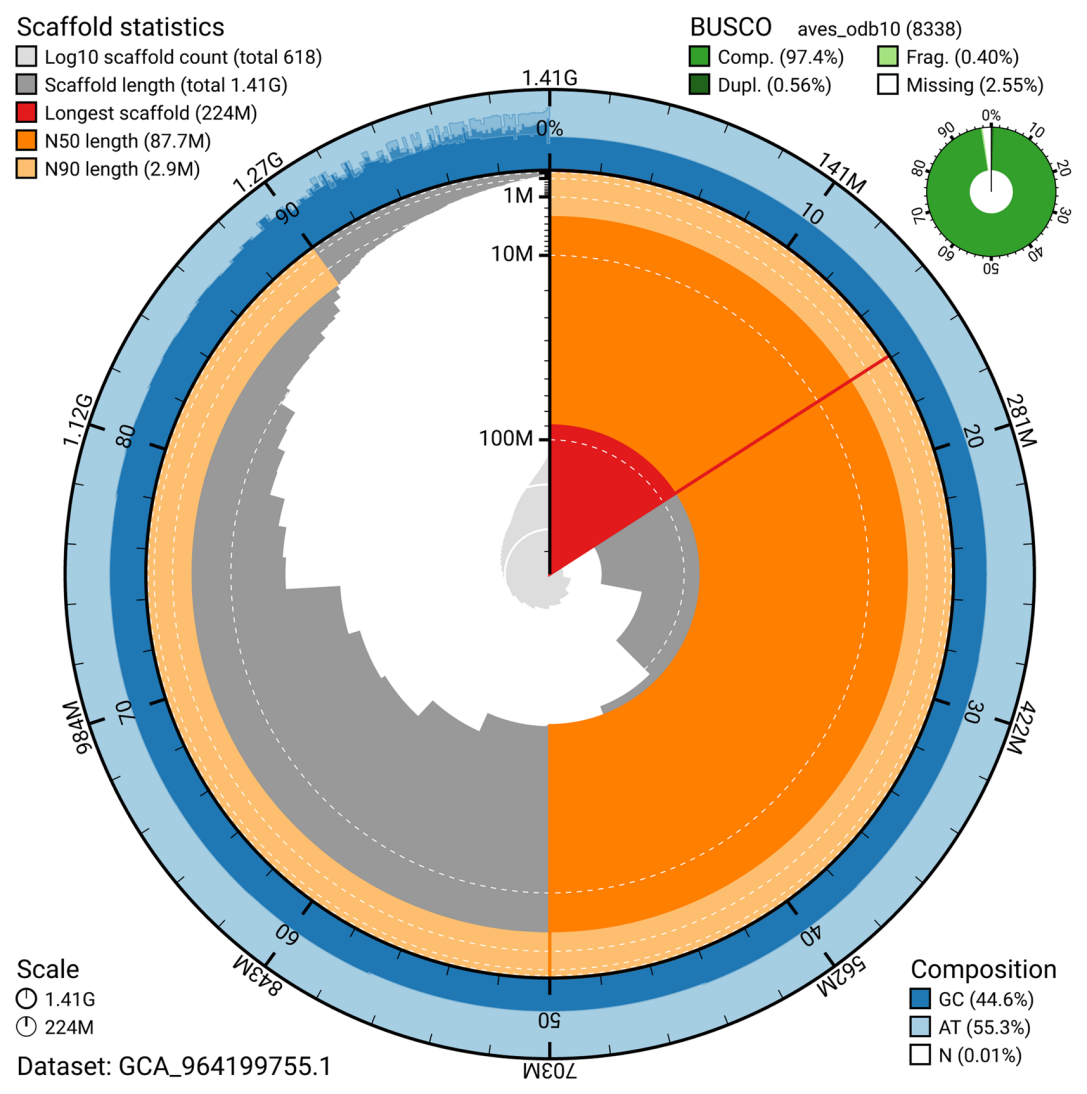
Genome assembly of *Larus michahellis*, bLarMic1.1: metrics. The BlobToolKit snail plot provides an overview of assembly metrics and BUSCO gene completeness. The circumference represents the length of the whole genome sequence, and the main plot is divided into 1,000 bins around the circumference. The outermost blue tracks display the distribution of GC, AT, and N percentages across the bins. Scaffolds are arranged clockwise from longest to shortest and are depicted in dark grey. The longest scaffold is indicated by the red arc, and the deeper orange and pale orange arcs represent the N50 and N90 lengths. A light grey spiral at the centre shows the cumulative scaffold count on a logarithmic scale. A summary of complete, fragmented, duplicated, and missing BUSCO genes in the aves_odb10 set is presented at the top right. An interactive version of this figure is available at https://blobtoolkit.genomehubs.org/view/GCA_964199755.1/dataset/GCA_964199755.1/snail.

**Figure 3.**
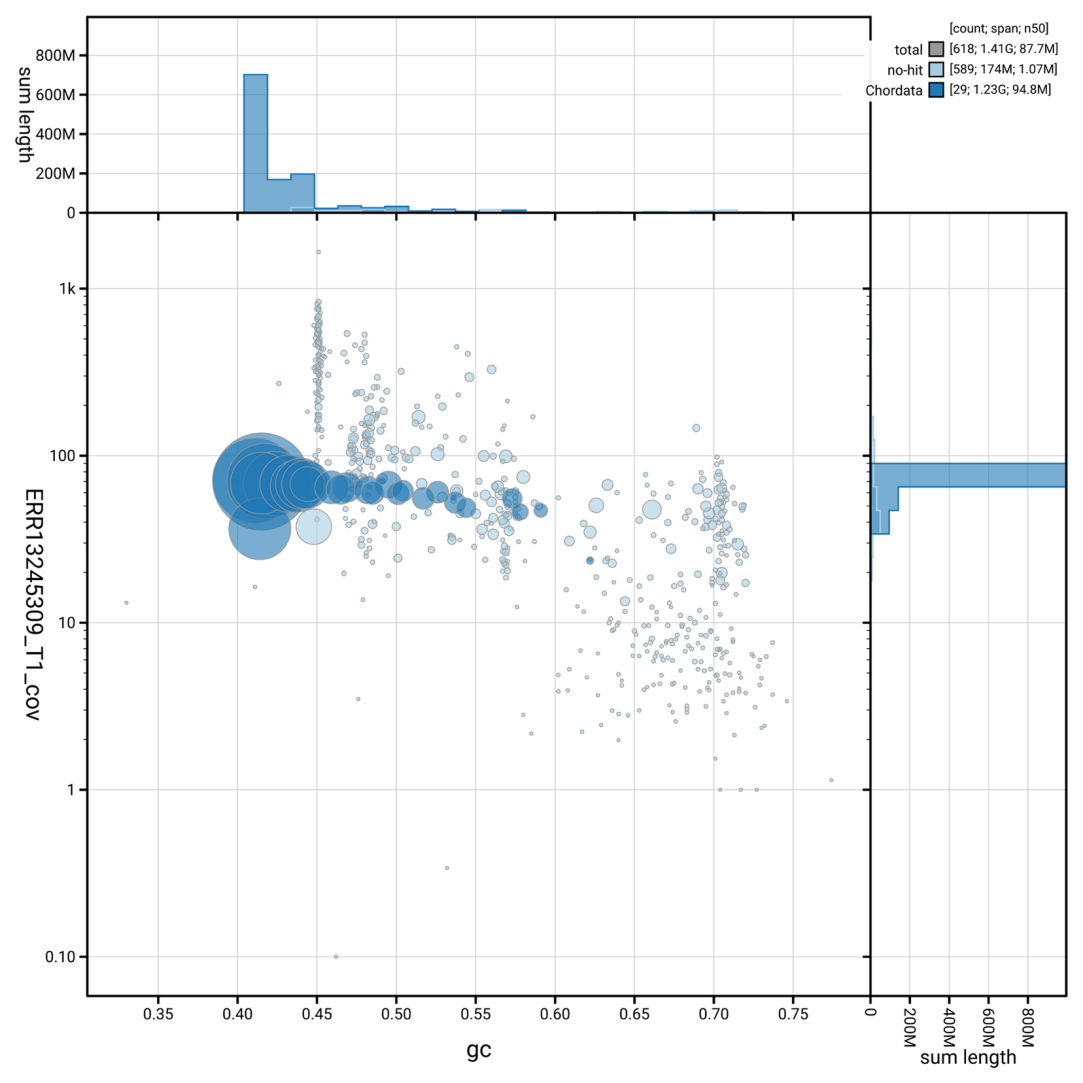
Genome assembly of *Larus michahellis*, bLarMic1.1: BlobToolKit GC-coverage plot. Blob plot showing sequence coverage (vertical axis) and GC content (horizontal axis). The circles represent scaffolds, with the size proportional to scaffold length and the colour representing phylum membership. The histograms along the axes display the total length of sequences distributed across different levels of coverage and GC content. An interactive version of this figure is available at https://blobtoolkit.genomehubs.org/view/GCA_964199755.1/blob.

**Figure 4.**
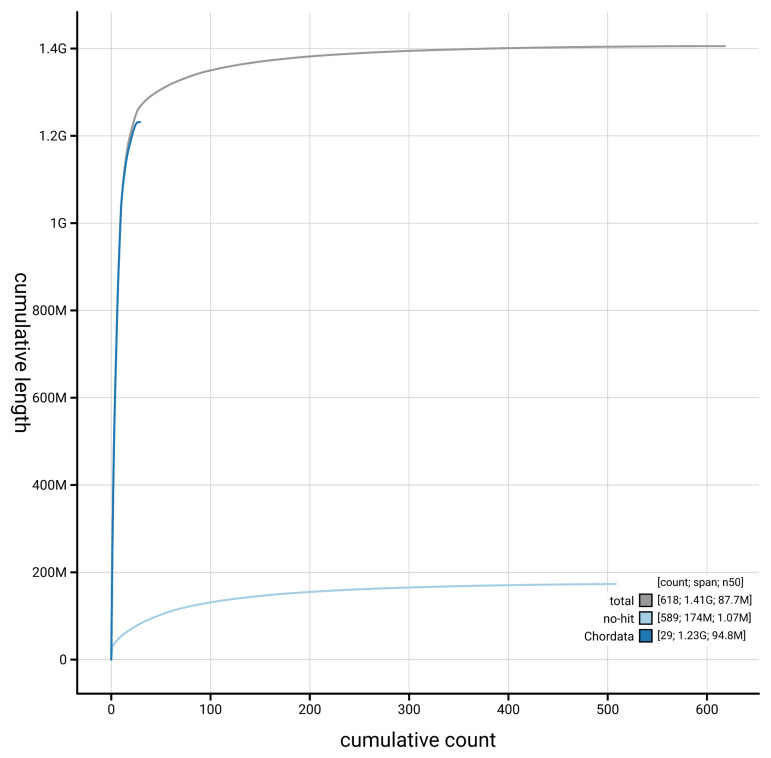
Genome assembly of *Larus michahellis,* bLarMic1.1: BlobToolKit cumulative sequence plot. The grey line shows cumulative length for all scaffolds. Coloured lines show cumulative lengths of scaffolds assigned to each phylum using the buscogenes taxrule. An interactive version of this figure is available at https://blobtoolkit.genomehubs.org/view/GCA_964199755.1/dataset/GCA_964199755.1/cumulative.

The snail plot in Figure 2 provides a summary of the assembly statistics, indicating the distribution of scaffold lengths and other assembly metrics. Figure 3 shows the distribution of scaffolds by GC proportion and coverage. Figure 4 presents a cumulative assembly plot, with separate curves representing different scaffold subsets assigned to various phyla, illustrating the completeness of the assembly.

**Figure 5.**
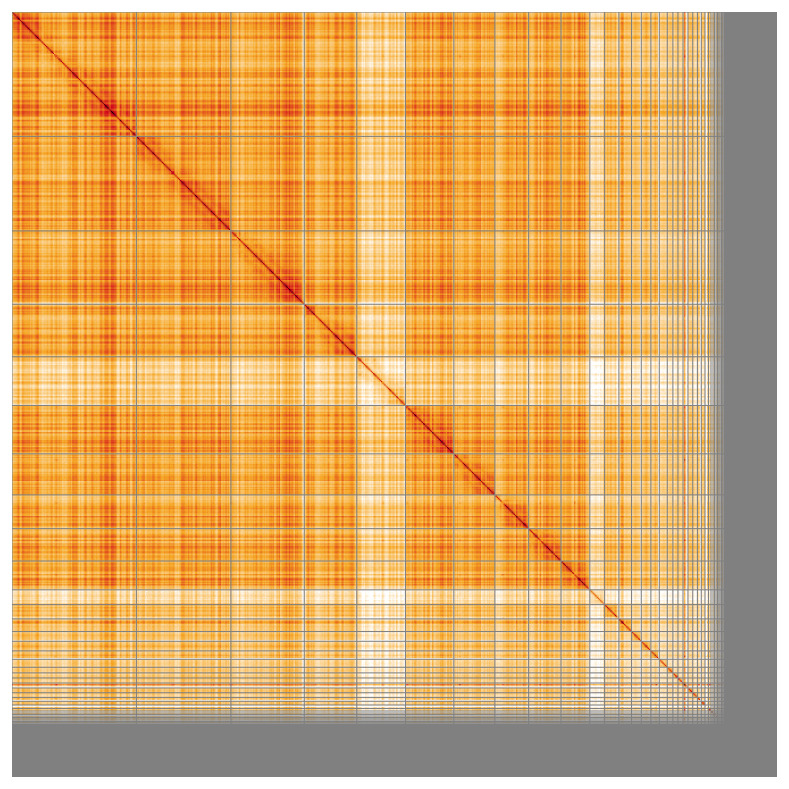
Genome assembly of *Larus michahellis*: Hi-C contact map of the bLarMic1.1 assembly, visualised using HiGlass. Chromosomes are shown in order of size from left to right and top to bottom. An interactive version of this figure may be viewed at https://genome-note-higlass.tol.sanger.ac.uk/l/?d=OircZnJzRdiOyEVdx1dciQ.

Most of the assembly sequence (90.15%) was assigned to 35 chromosomal-level scaffolds, representing 34 autosomes and the W and Z sex chromosome. These chromosome-level scaffolds, confirmed by Hi-C data, are named according to size ( Figure 5; Table 3). During curation, chromosomes Z and W were identified using read coverage.

**Table 3.** Chromosomal pseudomolecules in the genome assembly of *Larus michahellis*, bLarMic1.

INSDC accession	Name	Length (Mb)	GC%
OZ118746.1	1	224.38	41.5
OZ118747.1	2	170.44	41
OZ118748.1	3	132.37	41.5
OZ118749.1	4	94.79	42.5
OZ118751.1	5	87.29	41.5
OZ118752.1	6	73.96	43
OZ118753.1	7	60.95	43.5
OZ118754.1	8	58.36	44
OZ118755.1	9	51.87	44.5
OZ118757.1	10	25.64	44.5
OZ118758.1	11	22.76	46
OZ118759.1	12	17.67	47
OZ118760.1	13	17.39	46.5
OZ118761.1	14	14.99	48
OZ118762.1	15	14.45	49.5
OZ118763.1	16	10.19	48.5
OZ118764.1	17	9.13	50
OZ118765.1	18	9.02	52.5
OZ118766.1	19	8.98	51.5
OZ118767.1	20	8.52	50.5
OZ118768.1	21	8.42	53.5
OZ118769.1	22	7.03	57.5
OZ118770.1	23	6.85	54.5
OZ118771.1	24	4.52	58
OZ118772.1	25	3.44	62.5
OZ118773.1	26	2.9	59
OZ118774.1	27	1.59	55.5
OZ118775.1	28	1.3	53
OZ118776.1	29	1.26	61
OZ118777.1	30	1.04	64.5
OZ118778.1	31	0.49	62
OZ118779.1	32	0.32	53.5
OZ118780.1	33	0.13	69
OZ118756.1	W	26.87	45
OZ118750.1	Z	87.71	41.5
OZ118781.1	MT	0.02	45

The mitochondrial genome was also assembled. This sequence is included as a contig in the multifasta file of the genome submission and as a standalone record in GenBank.

### Assembly quality metrics

The estimated Quality Value (QV) and *k*-mer completeness metrics, along with BUSCO completeness scores, were calculated for each haplotype and the combined assembly. The QV reflects the base-level accuracy of the assembly, while *k*-mer completeness indicates the proportion of expected *k*-mers identified in the assembly. BUSCO scores provide a measure of completeness based on benchmarking universal single-copy orthologues.

The primary haplotype has a QV of 63.8, and the combined primary and alternate assemblies achieve an estimated QV of 63.9. The *k*-mer completeness for the primary haplotype is 94.01%, and for the alternate haplotype it is 84.30%. The combined primary and alternate assemblies achieve a *k*-mer completeness of 99.81%. BUSCO analysis using the aves_odb10 reference set ( *n* = 8,338) indicated a completeness score of 97.4% (single = 96.9%, duplicated = 0.6%).

Table 2 provides assembly metric benchmarks adapted from Rhie *et al.* (2021) and the Earth BioGenome Project (EBP) Report on Assembly Standards September 2024. The assembly achieves the EBP reference standard of **6.7.Q63**.

## Methods

### Sample acquisition

An adult female yellow-legged gull was captured with a walk-in trap on its nest on a rooftop at Kasthoferstrasse 42, 3006 Bern, Switzerland (46° 56′ 30.48″ N 7° 28′ 17.44″ E) on 18 April 2023, in accordance with capture and ringing permit BAFU-417.525-06-3/13/1 and animal experimentation permit BE129/2022. It was identified by David Marques, based on the features described above ( Figure 2) and on range, as *L. michahellis* is the only large gull species breeding in Switzerland. Standard measurements (weight: 990 g, tarsus: 63.6 mm, wing length: 445 mm, head length: 119.2 mm, beak length to feathering: 50.9 mm, beak depth at gonys: 18.6 mm, minimum beak depth: 17.1 mm), photographs ( Figure 2), a 200 µL blood sample from the leg vein was taken and a metal ring MA02451 fitted, followed by anaesthesia of the bird through blunt force trauma on the head and euthanasia by decapitation. The carcass was rapidly dissected and tissue samples from the brain, muscle, heart, liver, and oviduct were collected and immediately flash-frozen in liquid nitrogen. The rest of the bird is preserved as one wing preparation, a round skin and remaining tissue in ethanol in the bird collection of the Natural History Museum Basel, with accession NMB-AVES-23-001. Liquid nitrogen samples were stored at –80°C at the Natural History Museum Basel before being sent to the Wellcome Sanger Institute on dry ice.

Metadata collection for samples adhered to the Darwin Tree of Life project standards described by Lawniczak *et al*. (2022).

### Nucleic acid extraction

The workflow for high molecular weight (HMW) DNA extraction at the Wellcome Sanger Institute (WSI) Tree of Life Core Laboratory includes a sequence of procedures: sample preparation and homogenisation, DNA extraction, fragmentation and purification. Detailed protocols are available on protocols.io ( Denton *et al.*, 2023b). The bLarMic1 sample was prepared for DNA extraction by weighing and dissecting it on dry ice ( Jay *et al.*, 2023). Tissue from the oviduct was homogenised using a PowerMasher II tissue disruptor ( Denton *et al.*, 2023a). HMW DNA was extracted using the Automated MagAttract v2 protocol ( Oatley *et al.*, 2023a). DNA was sheared into an average fragment size of 12–20 kb in a Megaruptor 3 system ( Bates *et al.*, 2023). Sheared DNA was purified by solid-phase reversible immobilisation, using AMPure PB beads to eliminate shorter fragments and concentrate the DNA ( Oatley *et al.*, 2023b). The concentration of the sheared and purified DNA was assessed using a Nanodrop spectrophotometer and Qubit Fluorometer using the Qubit dsDNA High Sensitivity Assay kit. Fragment size distribution was evaluated by running the sample on the FemtoPulse system.

RNA was extracted from blood tissue of bLarMic1 in the Tree of Life Laboratory at the WSI using the RNA Extraction: Automated MagMax™ *mir*Vana protocol ( do Amaral *et al.*, 2023). The RNA concentration was assessed using a Nanodrop spectrophotometer and a Qubit Fluorometer using the Qubit RNA Broad-Range Assay kit. Analysis of the integrity of the RNA was done using the Agilent RNA 6000 Pico Kit and Eukaryotic Total RNA assay.

### Hi-C sample preparation

Tissue from the blood of the bLarMic1 sample was processed for Hi-C sequencing at the WSI Scientific Operations core, using the Arima-HiC v2 kit. In brief, 20–50 mg of frozen tissue (stored at –80 °C) was fixed, and the DNA crosslinked using a TC buffer with 22% formaldehyde concentration. After crosslinking, the tissue was homogenised using the Diagnocine Power Masher-II and BioMasher-II tubes and pestles. Following the Arima-HiC v2 kit manufacturer's instructions, crosslinked DNA was digested using a restriction enzyme master mix. The 5’-overhangs were filled in and labelled with biotinylated nucleotides and proximally ligated. An overnight incubation was carried out for enzymes to digest remaining proteins and for crosslinks to reverse. A clean up was performed with SPRIselect beads prior to library preparation. Additionally, the biotinylation percentage was estimated using the Qubit Fluorometer v4.0 (Thermo Fisher Scientific) and Qubit HS Assay Kit and Arima-HiC v2 QC beads.

### Library preparation and sequencing

Library preparation and sequencing were performed at the WSI Scientific Operations core.

### PacBio HiFi

At a minimum, samples were required to have an average fragment size exceeding 8 kb and a total mass over 400 ng to proceed to the low input SMRTbell Prep Kit 3.0 protocol (Pacific Biosciences, California, USA), depending on genome size and sequencing depth required. Libraries were prepared using the SMRTbell Prep Kit 3.0 (Pacific Biosciences, California, USA) as per the manufacturer's instructions. The kit includes the reagents required for end repair/A-tailing, adapter ligation, post-ligation SMRTbell bead cleanup, and nuclease treatment. Following the manufacturer’s instructions, size selection and clean up was carried out using diluted AMPure PB beads (Pacific Biosciences, California, USA). DNA concentration was quantified using the Qubit Fluorometer v4.0 (Thermo Fisher Scientific) with Qubit 1X dsDNA HS assay kit and the final library fragment size analysis was carried out using the Agilent Femto Pulse Automated Pulsed Field CE Instrument (Agilent Technologies) and gDNA 55kb BAC analysis kit.

Samples were sequenced on a Revio instrument (Pacific Biosciences, California, USA). Prepared libraries were normalised to 2 nM, and 15 μL was used for making complexes. Primers were annealed and polymerases were hybridised to create circularised complexes according to manufacturer’s instructions. The complexes were purified with the 1.2X clean up with SMRTbell beads. The purified complexes were then diluted to the Revio loading concentration (in the range 200–300 pM), and spiked with a Revio sequencing internal control. Samples were sequenced on Revio 25M SMRT cells (Pacific Biosciences, California, USA). The SMRT link software, a PacBio web-based end-to-end workflow manager, was used to set-up and monitor the run, as well as perform primary and secondary analysis of the data upon completion.

### Hi-C

For Hi-C library preparation, DNA was fragmented using the Covaris E220 sonicator (Covaris) and size selected using SPRISelect beads to 400 to 600 bp. The DNA was then enriched using the Arima-HiC v2 kit Enrichment beads. Using the NEBNext Ultra II DNA Library Prep Kit (New England Biolabs) for end repair, a-tailing, and adapter ligation. This uses a custom protocol which resembles the standard NEBNext Ultra II DNA Library Prep protocol but where library preparation occurs while DNA is bound to the Enrichment beads. For library amplification, 10 to 16 PCR cycles were required, determined by the sample biotinylation percentage. The Hi-C sequencing was performed using paired-end sequencing with a read length of 150 bp on an Illumina NovaSeq X instrument.

### RNA

Poly(A) RNA-Seq libraries were constructed using the NEB Ultra II RNA Library Prep kit, following the manufacturer’s instructions. RNA sequencing was performed on the Illumina NovaSeq X instrument.

### Genome assembly, curation and evaluation

***Assembly***

Prior to assembly of the PacBio HiFi reads, a database of *k*-mer counts ( *k* = 31) was generated from the filtered reads using FastK. GenomeScope2 ( Ranallo-Benavidez *et al.*, 2020) was used to analyse the *k*-mer frequency distributions, providing estimates of genome size, heterozygosity, and repeat content.

The HiFi reads were assembled using Hifiasm ( Cheng *et al.*, 2021) with the --primary option. Haplotypic duplications were identified and removed using purge_dups ( Guan *et al.*, 2020). The Hi-C reads were mapped to the primary contigs using bwa-mem2 ( Vasimuddin *et al.*, 2019). The contigs were further scaffolded using the provided Hi-C data ( Rao *et al.*, 2014) in YaHS ( Zhou *et al.*, 2023) using the --break option for handling potential misassemblies. The scaffolded assemblies were evaluated using Gfastats ( Formenti *et al.*, 2022), BUSCO ( Manni *et al.*, 2021) and MERQURY.FK ( Rhie *et al.*, 2020).

The mitochondrial genome was assembled using MitoHiFi ( Uliano-Silva *et al.*, 2023), which runs MitoFinder ( Allio *et al.*, 2020) and uses these annotations to select the final mitochondrial contig and to ensure the general quality of the sequence.

***Assembly curation***

The assembly was decontaminated using the Assembly Screen for Cobionts and Contaminants (ASCC) pipeline (article in preparation). Flat files and maps used in curation were generated in TreeVal ( Pointon *et al.*, 2023). Manual curation was primarily conducted using PretextView ( Harry, 2022), with additional insights provided by JBrowse2 ( Diesh *et al.*, 2023) and HiGlass ( Kerpedjiev *et al.*, 2018). Scaffolds were visually inspected and corrected as described by Howe *et al*. (2021). Any identified contamination, missed joins, and mis-joins were corrected, and duplicate sequences were tagged and removed. Sex chromosomes were identified by read coverage analysis. The curation process is documented at https://gitlab.com/wtsi-grit/rapid-curation (article in preparation).

***Assembly quality assessment***

The Merqury.FK tool ( Rhie *et al.*, 2020), run in a Singularity container ( Kurtzer *et al.*, 2017), was used to evaluate *k*-mer completeness and assembly quality for the primary and alternate haplotypes using the *k*-mer databases ( *k* = 31) that were computed prior to genome assembly. The analysis outputs included assembly QV scores and completeness statistics.

A Hi-C contact map was produced for the final version of the assembly. The Hi-C reads were aligned using bwa-mem2 ( Vasimuddin *et al.*, 2019) and the alignment files were combined using SAMtools ( Danecek *et al.*, 2021). The Hi-C alignments were converted into a contact map using BEDTools ( Quinlan & Hall, 2010) and the Cooler tool suite ( Abdennur & Mirny, 2020). The contact map is visualised in HiGlass ( Kerpedjiev *et al.*, 2018).

The blobtoolkit pipeline is a Nextflow port of the previous Snakemake Blobtoolkit pipeline ( Challis *et al.*, 2020). It aligns the PacBio reads in SAMtools and minimap2 ( Li, 2018) and generates coverage tracks for regions of fixed size. In parallel, it queries the GoaT database ( Challis *et al.*, 2023) to identify all matching BUSCO lineages to run BUSCO ( Manni *et al.*, 2021). For the three domain-level BUSCO lineages, the pipeline aligns the BUSCO genes to the UniProt Reference Proteomes database ( Bateman *et al.*, 2023) with DIAMOND blastp ( Buchfink *et al.*, 2021). The genome is also divided into chunks according to the density of the BUSCO genes from the closest taxonomic lineage, and each chunk is aligned to the UniProt Reference Proteomes database using DIAMOND blastx. Genome sequences without a hit are chunked using seqtk and aligned to the NT database with blastn ( Altschul *et al.*, 1990). The blobtools suite combines all these outputs into a blobdir for visualisation.

The blobtoolkit pipeline was developed using nf-core tooling ( Ewels *et al.*, 2020) and MultiQC ( Ewels *et al.*, 2016), relying on the Conda package manager, the Bioconda initiative ( Grüning *et al.*, 2018), the Biocontainers infrastructure ( da Veiga Leprevost *et al.*, 2017), as well as the Docker ( Merkel, 2014) and Singularity ( Kurtzer *et al.*, 2017) containerisation solutions.

Table 4 contains a list of relevant software tool versions and sources.

**Table 4.** Software tools: versions and sources

Software tool	Version	Source
BEDTools	2.30.0	https://github.com/arq5x/bedtools2
BLAST	2.14.0	ftp://ftp.ncbi.nlm.nih.gov/blast/executables/blast+/
BlobToolKit	4.3.9	https://github.com/blobtoolkit/blobtoolkit
BUSCO	5.5.0	https://gitlab.com/ezlab/busco
bwa-mem2	2.2.1	https://github.com/bwa-mem2/bwa-mem2
Cooler	0.8.11	https://github.com/open2c/cooler
DIAMOND	2.1.8	https://github.com/bbuchfink/diamond
fasta_windows	0.2.4	https://github.com/tolkit/fasta_windows
FastK	427104ea91c78c3b8b8b49f1a7d6bbeaa869ba1c	https://github.com/thegenemyers/FASTK
Gfastats	1.3.6	https://github.com/vgl-hub/gfastats
GoaT CLI	0.2.5	https://github.com/genomehubs/goat-cli
Hifiasm	0.19.8-r603	https://github.com/chhylp123/hifiasm
HiGlass	44086069ee7d4d3f6f3f0012569789ec138f42b84 aa44357826c0b6753eb28de	https://github.com/higlass/higlass
MerquryFK	d00d98157618f4e8d1a9190026b19b471055b22e	https://github.com/thegenemyers/MERQURY.FK
Minimap2	2.24-r1122	https://github.com/lh3/minimap2
MitoHiFi	3	https://github.com/marcelauliano/MitoHiFi
MultiQC	1.14, 1.17, and 1.18	https://github.com/MultiQC/MultiQC
NCBI Datasets	15.12.0	https://github.com/ncbi/datasets
Nextflow	23.10.0	https://github.com/nextflow-io/nextflow
PretextView	0.2	https://github.com/sanger-tol/PretextView
purge_dups	1.2.3	https://github.com/dfguan/purge_dups
samtools	1.19.2	https://github.com/samtools/samtools
sanger-tol/ascc	-	https://github.com/sanger-tol/ascc
sanger-tol/ blobtoolkit	0.5.1	https://github.com/sanger-tol/blobtoolkit
Seqtk	1.3	https://github.com/lh3/seqtk
Singularity	3.9.0	https://github.com/sylabs/singularity
TreeVal	1.2.0	https://github.com/sanger-tol/treeval
YaHS	1.2a.2	https://github.com/c-zhou/yahs

### Wellcome Sanger Institute – Legal and Governance

The materials that have contributed to this genome note have been supplied by a Darwin Tree of Life Partner. The submission of materials by a Darwin Tree of Life Partner is subject to the **‘Darwin Tree of Life Project Sampling Code of Practice’**, which can be found in full on the Darwin Tree of Life website here. By agreeing with and signing up to the Sampling Code of Practice, the Darwin Tree of Life Partner agrees they will meet the legal and ethical requirements and standards set out within this document in respect of all samples acquired for, and supplied to, the Darwin Tree of Life Project.

Further, the Wellcome Sanger Institute employs a process whereby due diligence is carried out proportionate to the nature of the materials themselves, and the circumstances under which they have been/are to be collected and provided for use. The purpose of this is to address and mitigate any potential legal and/or ethical implications of receipt and use of the materials as part of the research project, and to ensure that in doing so we align with best practice wherever possible. The overarching areas of consideration are:

•  Ethical review of provenance and sourcing of the material

•  Legality of collection, transfer and use (national and international)

Each transfer of samples is further undertaken according to a Research Collaboration Agreement or Material Transfer Agreement entered into by the Darwin Tree of Life Partner, Genome Research Limited (operating as the Wellcome Sanger Institute), and in some circumstances other Darwin Tree of Life collaborators.

## Data Availability

European Nucleotide Archive: Larus michahellis (yellow-legged gull). Accession number PRJEB76381; https://identifiers.org/ena.embl/PRJEB76381. The genome sequence is released openly for reuse. The *Larus michahellis* genome sequencing initiative is part of the Darwin Tree of Life (DToL) project (PRJEB40665) and the Vertebrate Genomes Project (PRJNA489243). All raw sequence data and the assembly have been deposited in INSDC databases. The genome will be annotated using available RNA-Seq data and presented through the Ensembl pipeline at the European Bioinformatics Institute. Raw data and assembly accession identifiers are reported in Table 1 and Table 2.

## Author information

Members of the Wellcome Sanger Institute Tree of Life Management, Samples and Laboratory team are listed here: https://doi.org/10.5281/zenodo.12162482.

Members of Wellcome Sanger Institute Scientific Operations: Sequencing Operations are listed here: https://doi.org/10.5281/zenodo.12165051.

Members of the Wellcome Sanger Institute Tree of Life Core Informatics team are listed here: https://doi.org/10.5281/zenodo.12160324.

Members of the Tree of Life Core Informatics collective are listed here: https://doi.org/10.5281/zenodo.12205391.

Members of the Darwin Tree of Life Consortium are listed here: https://doi.org/10.5281/zenodo.4783558.
