# Supplementary material for: The genome sequence of the Yellow-legged Gull, Larus michahellis Naumann, 1840
Source: Wellcome Open Res. 2025 Mar 14;10:129. [Version 1] doi: 10.12688/wellcomeopenres.23849.1 (PMC12082078; doi:10.12688/wellcomeopenres.23849.1)
Supplement: Peer review history

# Reviewer report: Reviewer response for version 1

**DOI:** 10.21956/wellcomeopenres.26309.r121330

**Published:** 2025-05-15

**Authors:** Zhou Wu

**Affiliations:** 1 The Roslin Institute, The University of Edinburgh, Midlothian, UK

This paper presents the genome assembly and gene annotation for the Yellow-legged Gull, which provides valuable genomic resources for future studies. The method of this paper is solid and the overall quality of the genome is good. The paper is also very well written, nicely documented all the details needed for the readers, I really enjoy reading it. Therefore, I only have some minor points that need to be clarified.

  

1. Please compare the genome assembly with the closely related species, focusing on aspects such as genomic statistics (e.g., repeat content) and chromosome topology (e.g., synteny blocks or alignment)
2. What were the criteria used for manual curation? Please briefly provide it.
3. It is unclear what was the RNA-sequencing data for, until I reached the Data availability section. The authors could mention this in the RNA section in Methods (Page 10 in PDF) that the data will be used for later Ensembl annotation.
4. Fig. 5: The HiC plot appears to show relatively weak chromosome structure definition. Could you clarify whether repetitive sequence filtering was implemented during the HiC map construction process? If so, please specify the methods and parameters used for this step.
5. Did the authors conduct repeat element detection analysis using tools like RepeatModeler and RepeatMasker?

Are sufficient details of methods and materials provided to allow replication by others?

Yes

Is the rationale for creating the dataset(s) clearly described?

Yes

Are the datasets clearly presented in a useable and accessible format?

Yes

Are the protocols appropriate and is the work technically sound?

Yes

Reviewer Expertise:

Avian genomics, bioinformatics, genome biology.

I confirm that I have read this submission and believe that I have an appropriate level of expertise to confirm that it is of an acceptable scientific standard, however I have significant reservations, as outlined above.

# Reviewer report: Reviewer response for version 1

**DOI:** 10.21956/wellcomeopenres.26309.r120748

**Published:** 2025-04-11

**Authors:** Xuejuan Li

**Affiliations:** 1 Shaanxi Normal University, Xi'an, Shaanxi, China

I have reviewed the manuscript entitled ‘The genome sequence of the Yellow-legged Gull, Larus michahellis Naumann, 1840’. In this study, the authors have assembled and annotated genome of the Yellow-legged Gull (Larus michahellis) at the chromosome level , with 35 scaffolds at the chromosomal level , genome size of 1,4 Gb, complete BUSCOs of 97.4%. The main comments are:

 1. Some detailed parameters need to be added in the methods, as different parameter setting may affect the results.

 2. The content of ‘Most of the assembly sequence (90.15%) was assigned to 35 chromosomal-level scaffolds, representing 34 autosomes and the W and Z sex chromosome.’ was in the ‘Assembly statistics’ section, but Table 2 contained only 33 autosomes, which was inconsistent.

 3. How are microchromosomes determined and disposed in the Larus michahellis assembly? Are there any dot chromosomes?

 4. The mitochondrial genome of Larus michahellis assembled with MitoHiFi contained only sequences but no annotation information (OZ118781). How do you avoid contamination of your mitochondrial genome assembly with nuclear copies of mitochondrial genes (NUMTs) sequences?

Are sufficient details of methods and materials provided to allow replication by others?

Yes

Is the rationale for creating the dataset(s) clearly described?

Yes

Are the datasets clearly presented in a useable and accessible format?

Yes

Are the protocols appropriate and is the work technically sound?

Yes

Reviewer Expertise:

avian genome; phylogeny; mitochondrial genome; evolution

I confirm that I have read this submission and believe that I have an appropriate level of expertise to confirm that it is of an acceptable scientific standard, however I have significant reservations, as outlined above.

# Reviewer report: Reviewer response for version 1

**DOI:** 10.21956/wellcomeopenres.26309.r121338

**Published:** 2025-04-11

**Authors:** Jochen B. W Wolf

**Affiliations:** 1 LMU Munich, Planegg-Martinsried, Germany

Ramos *et al* present a genome assembly of a heterogametic, female specimen of the Yellow-legged Gull. The generation of this genome is well motivated in the introduction. The Yellow-legged Gull is a taxon within a species complex with need for taxonomic revision which will be greatly aided by genome-wide data that can be placed on a reference. The assembly was done with state of the art methodology using long-read sequencing (PacBio Hifi) complemented with Hi-C for scaffolding and RNAseq for annotation. The data has been deposited to the NCBI database adhering to established standards. Quality metrics of the resulting assembly are presented in an understandable way and are guided by standard visuals (Figs.2-5). The methods section provides sufficient information, but it would be nice if the code used for all analyses should be made accessible. If I am not mistaken, only subsepta of the analyses are currently covered (e.g. assembly curation). Also, the authors may wish to wait until the genome is annotated through the ENSEMBL pipeline as announced in the Data Availability statement. Including core information on the annotation would, in my view, fit well within the scope of this paper.

Are sufficient details of methods and materials provided to allow replication by others?

Partly

Is the rationale for creating the dataset(s) clearly described?

Yes

Are the datasets clearly presented in a useable and accessible format?

Yes

Are the protocols appropriate and is the work technically sound?

Yes

Reviewer Expertise:

Avian genomics

I confirm that I have read this submission and believe that I have an appropriate level of expertise to confirm that it is of an acceptable scientific standard.
